# Chitin-Assisted Fabrication of an Fe_3_O_4_/BiOCl Composite for Visible-Light Photocatalytic Degradation of Ciprofloxacin

**DOI:** 10.3390/molecules31010134

**Published:** 2025-12-30

**Authors:** Xiaoxing Zeng, Kunlei Wang, Hongting Ye, Xiaofeng Gong, Yanhong Yao, Fei Feng

**Affiliations:** 1 School of Resources and Environment, Nanchang University, Nanchang 330031, China; xiaoxingzeng@ncu.edu.cn (X.Z.); wkl405@ncu.edu.cn (K.W.); xfgong@ncu.edu.cn (X.G.); yanhongyao@ncu.edu.cn (Y.Y.); 2 Nanchang City Urban Management and Comprehensive Law Enforcement Bureau, Nanchang 330031, China; 13037219155@163.com

**Keywords:** Fe_3_O_4_/chitin/BiOCl composite, photocatalytic activity, ciprofloxacin degradation, magnetic recoverability, photocatalytic stability

## Abstract

A novel recyclable composite, Fe_3_O_4_/chitin/BiOCl, was synthesized via a solvothermal approach using cetyltrimethylammonium bromide (CTAB), bismuth nitrate pentahydrate (Bi(NO_3_)_3_·5H_2_O), chitin, and Fe_3_O_4_ as precursors. The composite was systematically characterized via X-ray diffraction (XRD), scanning electron microscopy (SEM), transmission electron microscopy (TEM), Brunauer–Emmett-Teller (BET) analysis, ultraviolet–visible (UV-vis) spectroscopy, and vibrating sample magnetometry (VSM). Characterization results indicated that the incorporation of chitin significantly improved the porosity and specific surface area of the catalyst. Furthermore, the synergistic effects between chitin and Fe_3_O_4_ effectively reduced the recombination rate of photogenerated electron–hole pairs. The photocatalytic activity of the composite was evaluated by degrading ciprofloxacin (CIP) under visible-light irradiation. When the contents of Fe_3_O_4_ and chitin were 5% and 2% (by weight), respectively, the catalyst exhibited excellent photocatalytic performance with a degradation rate of 89.54%, and the rate constant was 5.1 times higher than that of pure BiOCl. Additionally, the catalyst exhibited excellent magnetic recoverability and photocatalytic stability.

## 1. Introduction

As industry and agriculture rapidly progress, organic pollutants such as persistent organic pollutants (POPs), dyes, endocrine-disrupting compounds (EDCs), and pharmaceutical residues have become the focus of environmental remediation efforts [1,2,3]. These contaminants are characterized by high chemical stability, strong bioaccumulation potential, and long-range transport capabilities, leading to their widespread distribution in aquatic environments, soil, and the atmosphere [1], causing irreversible damage to human health and ecosystems via food chain amplification. Among various wastewater treatment technologies, photocatalytic technology, due to its cost-effectiveness, lack of secondary pollution, and mild reaction conditions, is favored [4,5]. The performance of photocatalytic technology largely depends on the catalyst; thus, the synthesis of high-efficiency photocatalysts is a key research direction in current photocatalysis studies [6].

BiOCl, a typical layered bismuth-based photocatalytic material, has been widely applied in environmental remediation fields such as organic pollutant removal [7], photocatalytic CO_2_ reduction [8], and nitrogen fixation [9]. Its unique layered structure providessufficient interlayer space to facilitate atomic orbital polarization, thereby promoting efficient separation and migration of photogenerated charge carriers. This structure also endows BiOCl with pronounced exceptional ultraviolet (UV) light harvesting capabilities. However, BiOCl has a wide band gap (~3.2 eV), which limits its response to only UV light–accounting for approximately 4% of the solar spectrum. This severely restricts its practical application efficiency under visible light. Additionally, traditional powder-based BiOCl catalysts are difficult to recover efficiently after reactions, easily causing secondary pollution and increasing usage costs, which further hinders their industrial-scale application.

To enhance the visible-light photocatalytic activity of BiOCl, numerous strategies have been explored, such as semiconductor coupling [10], elemental doping [11], and morphological engineering [12], to extend its light-responsive spectrum. Meanwhile, magnetic components like Fe_3_O_4_ have been incorporated to enable efficient magnetic separation of the catalyst. Nevertheless, existing composite systems (e.g., those using graphene or carbon nanotubes as carriers) face significant limitations: high cost of carrier materials, complex preparation processes, insufficient biocompatibility, and the risk that magnetic components may shield the active sites of the catalyst or reduce the transport efficiency of photogenerated carriers.

Chitin, due to its rich functional groups, porous structure and environmentally friendly properties, has become an ideal choice for new catalyst carriers. As an abundant natural biopolymer from crustacean shells, chitin is biodegradable, low-cost, and non-toxic, avoiding secondary pollution of synthetic carriers. Structural merits: Its amino and hydroxyl groups chelate Fe^3+^/Bi^3+^ ions, promoting uniform dispersion of Fe_2_O_3_ and BiOCl nanoparticles and inhibiting agglomeration. Chitin’s functional groups adjust the composite’s electronic structure, enhance light absorption, and facilitate photogenerated electron–hole separation to boost activity. The three-dimensional network structure of chitin not only provides a high specific surface area to enhance the adsorption capacity of pollutants, but also, the hydroxyl and amino groups on its surface can act as electron transport channels, inhibiting the recombination of photogenerated electron–hole pairs [13,14]. Moreover, the introduction of Fe_3_O_4_ nanoparticles can endow the material with a magnetic recovery function and can form heterojunctions with BiOCl, further promoting carrier separation [15].

In this study, a ternary composite, Fe_3_O_4_/chitin/BiOCl, was constructed using a solvothermal method, utilizing biomass-derived chitin as a multifunctional matrix. By regulating the doping ratio of Fe_3_O_4_ and chitin, the morphology, band structure and magnetic response performance of the material can be controlled. This work systematically explored the promoting effect of the porous structure of chitin on the adsorption-catalysis synergy mechanism of pollutants, as well as the charge transfer path of the Fe_3_O_4_/chitin/BiOCl complex under visible-light excitation. Using ciprofloxacin (CIP) as a model persistent pollutant, CIP is a widely used fluoroquinolone antibiotic, frequently detected in water environments due to poor biodegradability, posing risks of antibiotic resistance gene spread. Its stable aromatic ring structure makes it hard to degrade via traditional methods, and its characteristic UV-vis absorption peaks enable convenient concentration monitoring, making it a classic model pollutant in photocatalysis. The visible-light catalytic activity and cycling stability of the composite catalyst were studied, and its degradation mechanism was employed through free radical trapping experiments.

## 2. Results and Discussion

### 2.1. Crystal Structure (XRD) Analysis

**Figure 1:**
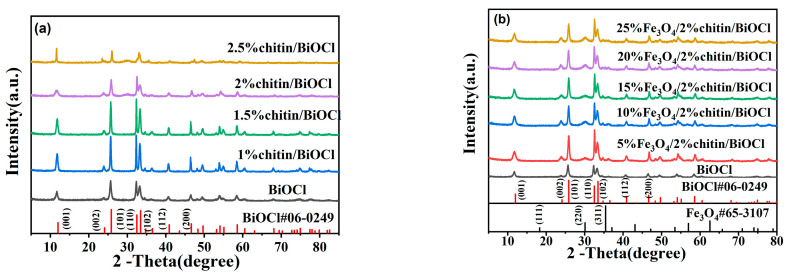
(**a**) XRD patterns of the obtained samples of BiOCl and (**b**) chitin/BiOCl; Fe_3_O_4_/chitin/BiOCl drawn.

The XRD patterns of the synthesized catalysts are depicted in Figure 1. As presented in Figure 1a, all diffraction peaks for catalysts with or without chitin incorporation align well with the tetragonal phase of BiOCl (JCPDS #06-0249), and no impurity peaks are observed, confirming the high crystallinity and phase purity of the BiOCl products. Distinct diffraction peaks at 2θ = 11.81°, 23.88°, 25.85°, 32.52°, 33.34°, 40.85°, and 46.72° correspond to the (001), (002), (101), (110), (102), (112), and (200) crystallographic planes of tetragonal BiOCl, respectively. Notably, the intensity of these peaks gradually increased with elevated chitin content, suggesting enhanced crystallinity. However, when the chitin content reached 2.0 wt%, a reduction in peak intensity accompanied by peak broadening emerged (Figure 1b), indicative of decreased crystallite size along specific lattice orientations. This observation implies that chitin modulates the crystal growth direction of BiOCl, likely by interacting with specific facets during hydrothermal synthesis.

As shown in Figure 1b, a new diffraction peak emerged at 2θ = 30.1°, which matches the (304) crystallographic plane of magnetite (Fe_3_O_4_, JCPDS #65-3107), confirming the successful incorporation of Fe_3_O_4_ into the composite. The intensity of this characteristic Fe_3_O_4_ peak progressively increased with higher Fe_3_O_4_ loading, as depicted in the magnified XRD profile (Figure 1b, inset). Furthermore, the Fe_3_O_4_/chitin/BiOCl composites displayed pronounced diffraction peaks, suggesting relatively large crystallite sizes compared to pure BiOCl.

No characteristic peaks of chitin were detected in any of the samples, which may be attributed to two factors: (1) the low concentration of chitin (≤2.5 wt%), and (2) the amorphous nature of chitin due to its non-metallic composition. Importantly, the diffraction peak positions of chitin/BiOCl and Fe_3_O_4_/chitin/BiOCl composites were identical to those of pure BiOCl, indicating that neither chitin nor Fe_3_O_4_ changed the tetragonal crystal structure of BiOCl. The absence of peak shifts or impurity phases further supports the high phase purity of the synthesized composites.

### 2.2. Morphological Analysis (SEM and TEM)

**Figure 2:**
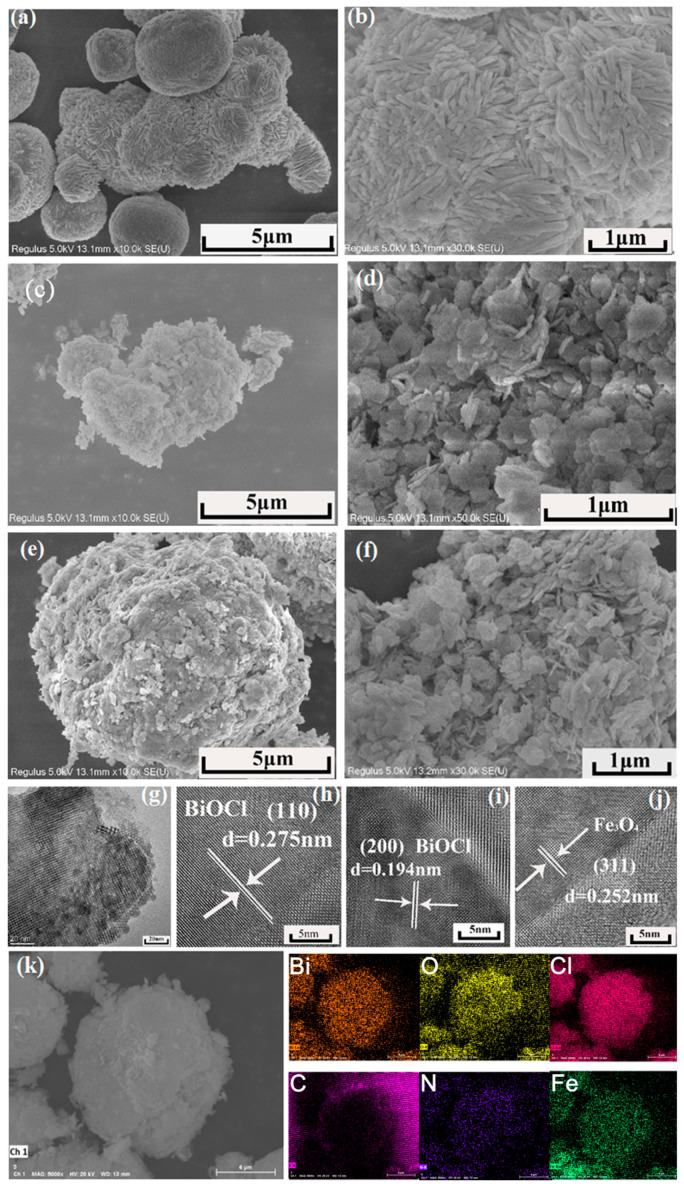
Morphological Analysis (SEM and TEM) of BiOCl samples. (**a**–**f**) the SEM of samples, (**g**–**j**) TEM analysis of BiOCl samples, and (**k**) the EDS analysis.

The morphological features of the catalysts are illustrated in Figure 2. Pure BiOCl exhibits a hierarchical structure composed of 2–3 μm nanospheres, which are densely stacked by numerous nanosheets (Figure 2a–c). Upon chitin incorporation, the BiOCl architecture transitions into irregular microspheres with interparticle voids and a fluffy, porous surface, forming a loosely packed flower-like morphology (Figure 2d–f). For the 5% Fe_3_O_4_/2%chitin/BiOCl composite, SEM images (Figure 2g–i) reveal uniform microspheres (5–10 μm) with compact and well-defined shapes, contrasting sharply with the more disordered structure of 2%chitin/BiOCl (Figure 2d–f). This structural densification is attributed to the aggregation effect induced by Fe_3_O_4_ nanoparticles, while chitin maintains the material’s porosity and enhances its specific surface area.

TEM and HRTEM analyses of 5%Fe_3_O_4_/2% chitin/BiOCl further elucidate its microstructure (Figure 2g–k). The TEM image (Figure 2g) confirms the coexistence of ultrathin BiOCl nanosheets and Fe_3_O_4_ nanoparticles anchored on their surfaces. HRTEM (Figure 2h,i) reveals distinct lattice fringes with d-spacings of 0.275 nm and 0.19 nm, corresponding to the (110) and (200) planes of tetragonal BiOCl, respectively. Additionally, from Figure 2j, a d-spacing of 0.252 nm aligns with the (311) plane of cubic Fe_3_O_4_ (JCPDS #65-3107), corroborating its successful integration.

Energy-dispersive X-ray spectroscopy (EDS) mapping (Figure 2k) confirms the uniform distribution of C, N, O, Bi, Cl, and Fe throughout the composite—consistent with the chemical composition of chitin and Fe_3_O_4_. Collectively, these results validate the effective incorporation of both chitin and Fe_3_O_4_ into the BiOCl matrix.

### 2.3. Surface Area (BET) Analysis

**Figure 3:**
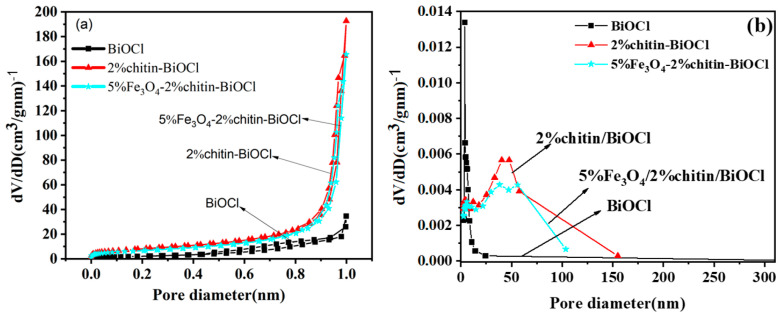
N_2_ adsorption–desorption isotherms (**a**) and pore distribution (**b**) of BiOCl, 2%chitin/BiOCl, 5%Fe_3_O_4_/2%chitin/BiOCl.

The N_2_ adsorption–desorption isotherms and pore size distributions of BiOCl, 2% chitin/BiOCl, and 5%Fe_3_O_4_/2%chitin/BiOCl are illustrated in Figure 3a. According to the IUPAC classification for mesoporous materials [16], all three catalysts could be unambiguously classified as exhibiting typical Type IV adsorption–desorption isotherms. Furthermore, the hysteresis loops of 2% chitin/BiOCl and 5%Fe_3_O_4_/2%chitin/BiOCl display an H3-type pattern, suggesting the presence of non-uniform slit-shaped pores within the samples. Notably, the isotherm profiles of 2%chitin/BiOCl and 5%Fe_3_O_4_/2%chitin/BiOCl are highly similar, confirming their analogous porous structures (Figure 3a), which aligns with the SEM observations.

The specific surface areas of BiOCl, 2%chitin/BiOCl, and 5%Fe_3_O_4_/2%chitin/BiOCl were determined to be 7.1065, 28.2142, and 22.9057 cm^3^·g^−1^, respectively. As shown in Figure 3b, the pore size distribution of 5%Fe_3_O_4_/2%chitin/BiOCl is predominantly skewed toward meso/macropores. These results demonstrate that the incorporation of chitin effectively enhances both the specific surface area and pore size of the material. A larger surface area and pore structure can provide abundant active sites for photochemical reactions and facilitate pollutant adsorption, thereby improving photocatalytic performance. The slight decrease in the specific surface area of 5%Fe_3_O_4_/2%chitin/BiOCl compared to that of 2% chitin/BiOCl might be attributed to the loading of Fe_3_O_4_ nanoparticles, which could partially reduce the surface area.

### 2.4. XPS Analysis

**Figure 4:**
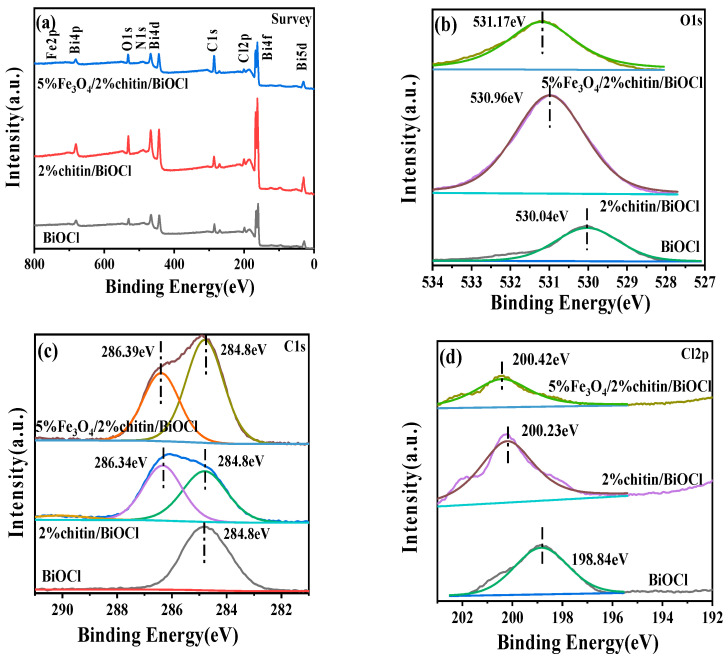

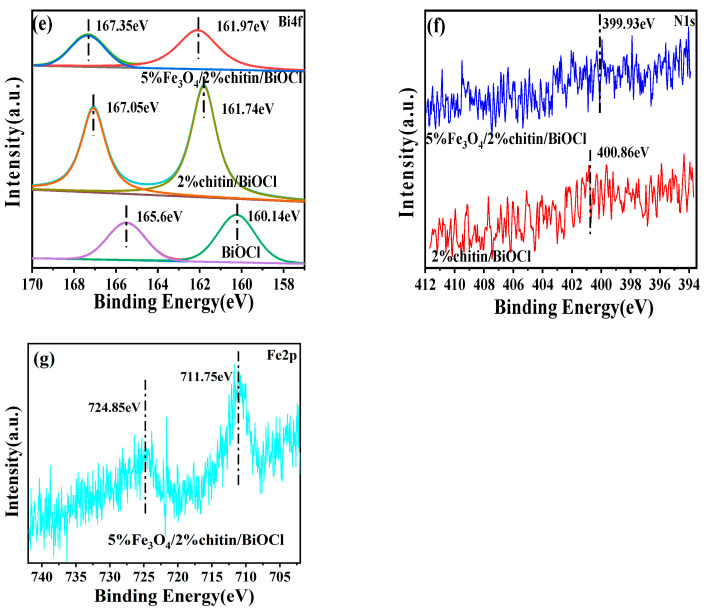
Survey (**a**), O1s (**b**), C1s (**c**), Cl2p (**d**), Bi4f (**e**), N1s (**f**), Fe2p (**g**) XPS spectra of the obtained samples BiOCl, 2%chitin/BiOCl, 5%Fe_3_O_4_/2%chitin/BiOCl.

The elemental composition and chemical states of the composites were analyzed in detail by X-ray photoelectron spectroscopy (XPS). Figure 4a presents the full-scan spectrum of 5%Fe_3_O_4_/2%chitin/BiOCl, confirming the coexistence of C, O, Bi, Cl, N, and Fe. Deconvoluted O 1s spectra of the samples (Figure 4b) reveal a dominant peak at 531.17 eV, which corresponds to lattice oxygen in Bi-O bonds within the [Bi_2_O_2_]^2+^ layers of BiOCl [17]. The Cl 2p3/2 peak at 200.44 eV is assigned to Cl^−^ in the composite [18]. Two distinct C 1s peaks are observed: the signal at 286.39 eV originates from carbon in chitin, while the lower-binding-energy peak at 284.8 eV arises from adventitious carbon contamination during XPS measurement [19]. The sharp doublet peaks at 161.97 eV (Bi 4f7/2) and 167.35 eV (Bi 4f5/2) confirm the presence of Bi^3+^ oxidation states in the composite [20]. A distinct N 1s peak at 399.93 eV further verifies the retention of nitrogen from chitin. Notably, a prominent Fe 2p peak at 711.75 eV unambiguously demonstrates the successful integration of Fe_3_O_4_. These XPS signatures collectively confirm the coexistence of all designed components in the composite photocatalyst, consistent with the XRD data.

The elemental composition and chemical states of the synthesized composites were systematically investigated by X-ray photoelectron spectroscopy (XPS). Figure 4a displays the full-scan XPS spectrum of the 5%Fe_3_O_4_/2%chitin/BiOCl composite, which confirms the coexistence of six constituent elements: carbon (C), oxygen (O), bismuth (Bi), chlorine (Cl), nitrogen (N), and iron (Fe). High-resolution spectral deconvolution provides further insight into the chemical bonding states of individual elements.

The O 1s spectrum (Figure 4b) exhibits a predominant peak centered at 531.17 eV, characteristic of lattice oxygen in Bi-O bonds within the [Bi_2_O_2_]^2+^ structural layers of BiOCl [17]. Chlorine species are identified through the Cl 2p3/2 peak at 200.44 eV, corresponding to Cl^−^ anions in the BiOCl matrix [18]. Carbon speciation analysis reveals two distinct contributions: the primary C 1s peak at 286.39 eV originates from chitin-derived carbon, while the secondary peak at 284.8 eV is attributed to adventitious carbon contamination inherent to XPS measurements [19].

Bismuth oxidation states were confirmed through well-resolved doublet peaks at 161.97 eV (Bi 4f7/2) and 167.35 eV (Bi 4f5/2), verifying the predominant Bi^3+^ state [20]. The preservation of chitin’s nitrogen content is evidenced by the N 1s signal at 399.93 eV. Notably, the Fe 2p spectrum displays a characteristic peak at 711.75 eV, providing definitive evidence for successful Fe_3_O_4_ incorporation. The systematic XPS characterizations confirm the successful incorporation of all constituent elements in the composite photocatalyst, with the chemical state profiles demonstrating remarkable consistency with the crystalline phase composition revealed by corresponding XRD patterns.

### 2.5. FT-IR Analysis

**Figure 5:**
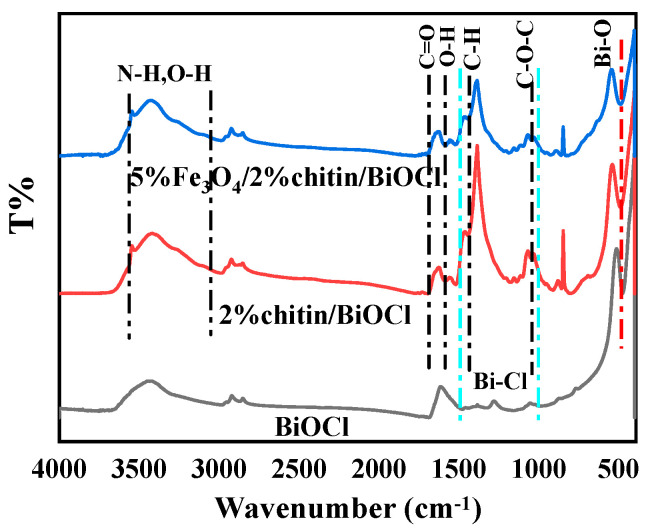
FT-IR spectra of BiOCl, 2%chitin/BiOCl, 5%Fe_3_O_4_/2%chitin/BiOCl.

The chemical functionalities of the composites were investigated by FT-IR spectroscopy (Figure 5). The infrared spectra display characteristic absorption bands corresponding to all constituent elements. A distinct O-H bending vibration is observed at 1602 cm^−1^, while the broad absorption envelope spanning 2900–3600 cm^−1^ arises from overlapping N-H/O-H stretching vibrations at the surface of the 5%Fe_3_O_4_/2%chitin/BiOCl ternary composite [21]. The Bi-O lattice vibration manifests as a characteristic peak at 505 cm^−1^, with additional Bi-Cl stretching modes identified in the 1000–1500 cm^−1^ region [22]. In the BiOCl/chitin binary composite, two new vibrational signatures emerge: a C-H bending mode at 1429 cm^−1^ [19] and a C-H stretching vibration at 2723 cm^−1^ [23], both originating from chitin integration. Furthermore, the presence of chitin is confirmed by diagnostic C-O-C (1062 cm^−1^) and C=O (1729 cm^−1^) stretching vibrations [24]. These spectral evidences collectively validate the structural preservation of chitin within the composite system.

### 2.6. UV-Vis Diffuse Reflectance Spectroscopy Analysis

**Figure 6:**
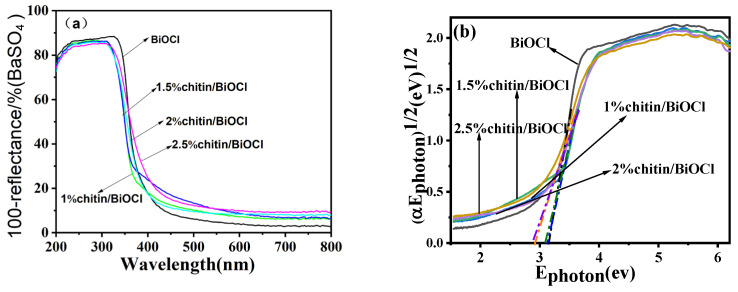

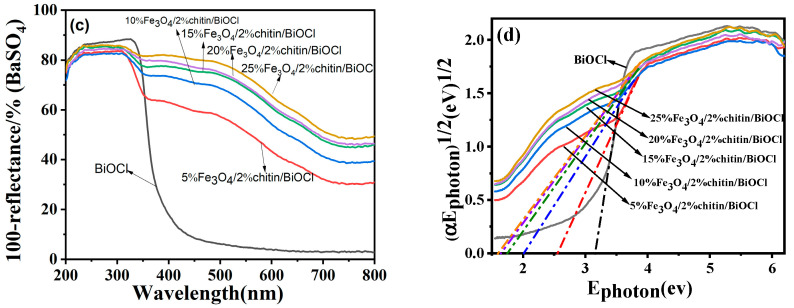
(**a**,**c**) UV–Vis DRS absorption spectra, (**b,d**) (α·Ephoton)^1/2^—Ephoton curves of the BiOCl, chitin/BiOCl, Fe_3_O_4_/chitin/BiOCl.

The UV-Vis diffuse reflectance spectra (DRS) of the synthesized catalysts are displayed in Figure 6. As shown in Figure 6a, the incorporation of chitin induces a red-shift in the absorption edge of the catalyst toward the visible-light region. Notably, the introduction of Fe_3_O_4_ further expands the light absorption range of the composite, as evidenced by the enhanced absorption intensity across both UV and visible regions (Figure 6c). The bandgap energies of the samples, calculated via the Tauc plot extrapolation method, are summarized in Figure 6b,d. pure BiOCl exhibits a bandgap of 3.2 eV, confirming its limited photoactivity under UV irradiation. In contrast, the chitin- and Fe_3_O_4_-modified BiOCl composites demonstrate significant red-shifted absorption edges and markedly intensified visible-light absorption, effectively narrowing their bandgaps to 2.8 eV (2%chitin/BiOCl) and 2.5 eV (5%Fe_3_O_4_/2%chitin/BiOCl), respectively. These results highlight the synergistic role of chitin and Fe_3_O_4_ in optimizing the optical absorption properties of BiOCl for visible-light-driven photocatalysis.

### 2.7. Photoluminescence (PL) Spectra Analysis

**Figure 7:**
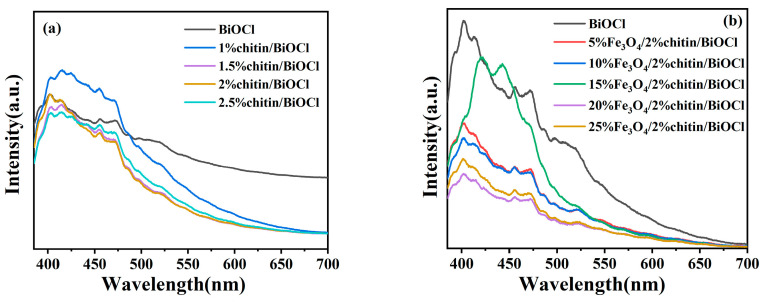
(**a**) PL spectra of BiOCl, chitin/BiOCl and, (**b**) for the Fe_3_O_4_/chitin/BiOCl.

Photoluminescence (PL) spectroscopy was employed to evaluate the migration and recombination dynamics of photogenerated electron–hole pairs in the catalysts. A lower PL emission intensity typically indicates suppressed charge recombination, which correlates with enhanced photocatalytic activity [25]. As depicted in Figure 7, the PL spectra of the obtained catalysts were recorded under an excitation wavelength of 360 nm. Notably, both chitin/BiOCl and Fe_3_O_4_/chitin/BiOCl composites exhibit significantly weaker PL intensities compared to the pure BiOCl, with the 2%chitin/BiOCl sample showing the weakest emission. This trend confirms that chitin incorporation effectively suppresses the recombination of photogenerated carriers. Furthermore, the additional introduction of Fe_3_O_4_ leads to a further reduction in PL intensity, demonstrating that the synergistic interaction between chitin and Fe_3_O_4_ markedly inhibits electron–hole recombination. Such inhibition facilitates efficient charge separation and migration, thereby amplifying the photocatalytic efficiency of the composite system.

### 2.8. VSM Analysis

**Figure 8:**
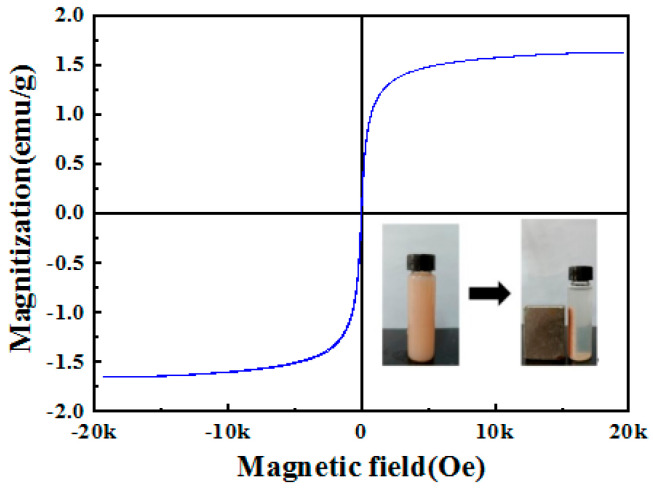
Magnetization curves of the obtained samples 5%Fe_3_O_4_/2%chitin/BiOCl. Inset: a photograph showing magnetic recycling of the 5%Fe_3_O_4_/2%chitin/BiOCl magnetic photocatalyst.

The magnetic properties of 5%Fe_3_O_4_/2%chitin/BiOCl were characterized using a vibrating sample magnetometer (VSM) to confirm its magnetic recyclability (Figure 8). The composite exhibits excellent super para magnetism, as evidenced by its S-shaped hysteresis loop with negligible coercivity and remanence, attributed to the presence of Fe_3_O_4_ nanoparticles. This magnetic responsiveness enables rapid separation of the photocatalyst from the reaction solution within minutes under an external magnetic field. Furthermore, the facile magnetic recovery of 5%Fe_3_O_4_/2%chitin/BiOCl facilitates efficient reuse in cyclic environments.

### 2.9. Photocatalytic Activity Analysis

**Figure 9:**
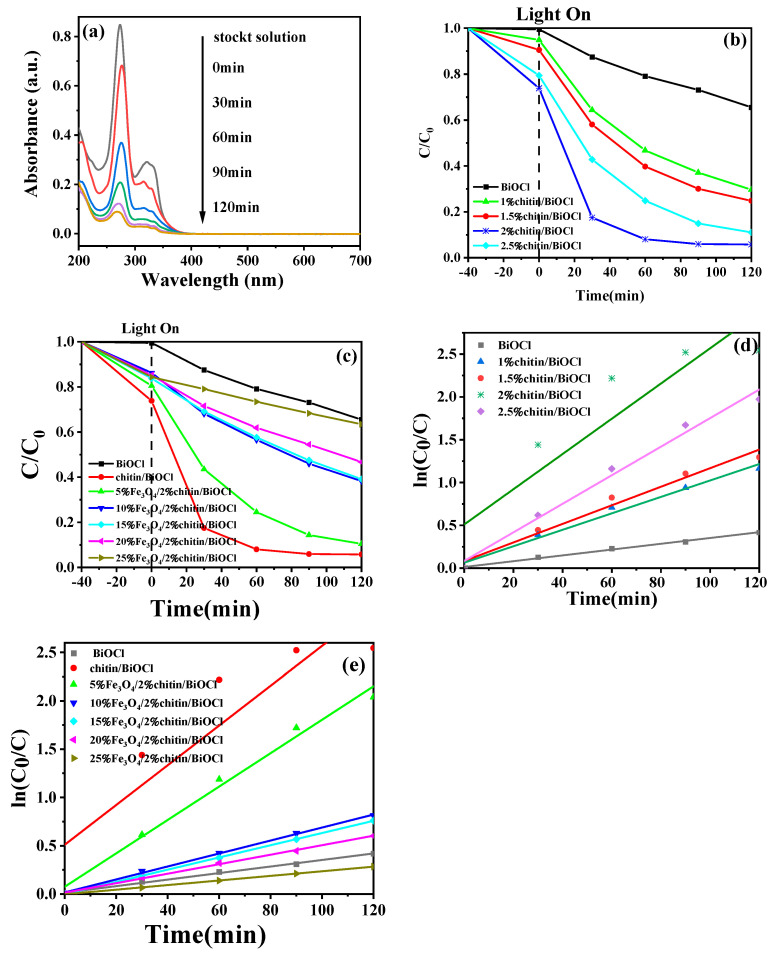
(**a**) UV-vis absorption spectral changes of CIP; photocatalytic performance for CIP solution under visible-light irradiation of catalysts obtained (**b**) chitin/BiOCl and (**c**) Fe_3_O_4_/chitin/BiOCl; plots of ln(C_0_/C) as a function of visible-light irradiation time (t) for photodegradation of CIP solution containing of catalysts obtained (**d**) chitin/BiOCl and (**e**) Fe_3_O_4_/chitin/BiOCl.

The photocatalytic performance of the catalysts was evaluated by degrading ciprofloxacin (CIP, 10 mg·L^−1^) under visible-light irradiation. Figure 9a displays the time-dependent UV-Vis absorption spectra of CIP during degradation by 5%Fe_3_O_4_/2%chitin/BiOCl, showing a progressive attenuation of the characteristic CIP peak at 276 nm. Analogous spectral trends were observed for all catalysts. Prior to irradiation, a 40-min dark adsorption step ensured adsorption–desorption equilibrium between CIP and the catalysts.

As illustrated in Figure 9, pristine BiOCl exhibited negligible adsorption (0.48%), whereas chitin-modified catalysts (1–2.5% chitin) demonstrated significantly enhanced adsorption capacities (5.08%, 9.44%, 26.15%, and 20.56%, respectively). This confirms chitin’s role in improving adsorption, though excessive chitin (>2%) reduced degradation efficiency due to active site blockage. The 2% chitin/BiOCl composite achieved the highest photocatalytic degradation efficiency (94.21%), surpassing pristine BiOCl (34.49%) by 2.7-fold. Similarly, Fe_3_O_4_ incorporation enhanced performance, with 5%Fe_3_O_4_/2%chitin/BiOCl reaching 89.54% degradation. Higher Fe_3_O_4_ loadings diminished efficiency, attributed to light shielding by magnetic Fe_3_O_4_ and reduced BiOCl content. Thus, 5% Fe_3_O_4_ was identified as the optimal loading to balance photocatalytic activity and magnetic recoverability.

The degradation kinetics followed pseudo-first-order behavior (Figure 9d,e). The 2% chitin/BiOCl composite exhibited the highest rate constant (0.02057 min^−1^), 6.1 times greater than pristine BiOCl (0.00339 min^−1^). For Fe_3_O_4_-modified catalysts, the 5%Fe_3_O_4_/2%chitin/BiOCl composite showed a rate constant of 0.01729 min^−1^, representing a 5.1-fold improvement over BiOCl. These results highlight the synergistic effects of chitin and Fe_3_O_4_ in optimizing charge separation and reaction kinetics.

### 2.10. Active Species and Mechanistic Analysis

**Figure 10:**
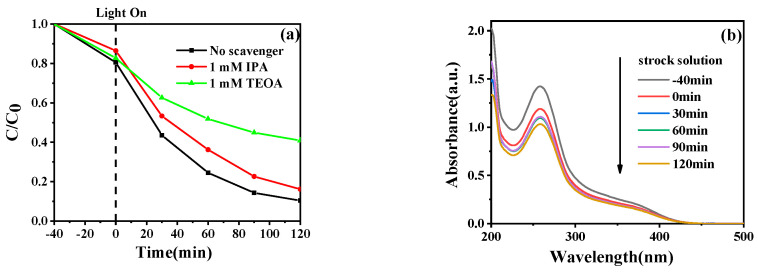
(**a**) Photo-degradation performance for CIP solution by 5%Fe_3_O_4_/2%chitin/BiOCl in the presence of various radical scavengers and (**b**) UV-vis absorption spectra of NBT in the 5%Fe_3_O_4_/2%chitin/BiOCl suspension.

To identify the dominant reactive species in the photocatalytic degradation process, radical trapping experiments were conducted using isopropanol (IPA, ·OH scavenger), triethanolamine (TEOA, h^+^ scavenger), and nitroblue tetrazolium chloride (NBT, ·O_2_^−^ scavenger). As shown in Figure 10a, the CIP degradation efficiency by 5%Fe_3_O_4_/2%chitin/BiOCl decreased from 89.54% (no scavenger) to 83.79% and 59.06% upon addition of IPA and TEOA, respectively. The marginal inhibition by IPA suggests limited ·OH involvement, whereas the significant efficiency drop with TEOA confirms photogenerated holes (h^+^) as the primary active species.

The generation of ·O_2_^−^ was verified by monitoring NBT degradation under visible light (Figure 10b). The gradual attenuation of NBT’s characteristic absorption peak at 259 nm over time confirms sustained ·O_2_^−^ production. Collectively, these results establish that h^+^, ·OH, and ·O_2_^−^ collaboratively drive the photocatalytic degradation, with h^+^ playing the predominant role.

### 2.11. Photocatalytic Mechanism

Under visible-light irradiation, the Fe_3_O_4_/chitin/BiOCl catalyst generates photogenerated holes (h^+^) and electrons (e^−^) (Equation (1)). The electrons subsequently reduce adsorbed O_2_ to form superoxide radicals (·O_2_^−^) (Equation (2)), while water molecules are oxidized by h^+^ to yield hydroxyl radicals (·OH) and protons (Equation (3)). The adsorbed CIP molecules are then degraded into CO_2_, H_2_O, and small organic intermediates via oxidation by h^+^, ·OH, and ·O_2_^−^ (Equation (4)).

Fe_3_O_4_/chitin/BiOCl + hν → h^+^ + e^−^ (1)

O_2_ + e^−^ → ·O_2_^−^ (2)

H_2_O + h^+^ → ·OH + H^+^ (3)

CIP + h^+^ + ·O_2_^−^ + ·OH → CO_2_ + H_2_O + intermediates (4)

### 2.12. Reusability and Stability Analysis

**Figure 11:**
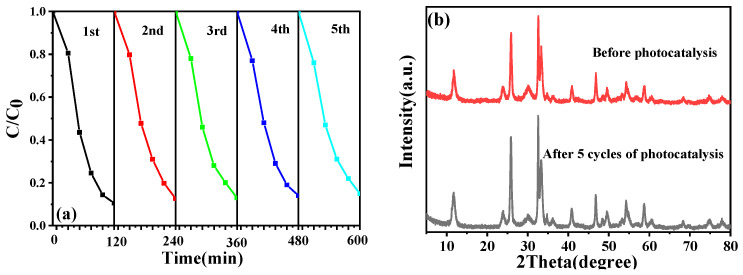
(**a**) CIP degradation efficiency and (**b**) XRD patterns of BiOCl.

The photocatalytic stability of 5%Fe_3_O_4_/2%chitin/BiOCl, a critical parameter for practical applications, was evaluated through cyclic degradation experiments under visible light. After each cycle, the catalyst was magnetically recovered, washed, dried at 60 °C for 6 h, and reused. As shown in Figure 11a, the CIP degradation efficiency remained stable at ~85% even after five consecutive cycles, with a minor decline attributed to partial catalyst loss during recovery.

XRD patterns (Figure 11b) reveal identical characteristic diffraction peaks, confirming its structural integrity. These results demonstrate the robust long-term stability of Fe_3_O_4_/chitin/BiOCl, underscoring its sustainability for repeated photocatalytic degradation of organic pollutants.

## 3. Experimental Section

### 3.1. Materials and Chemicals

All chemicals used in this study were of analytical grade (AR) unless otherwise specified, and no further purification was performed. Potassium chloride (KCl), bismuth nitrate pentahydrate (Bi(NO_3_)_3_·5H_2_O), and ethylene glycol (EG) were purchased from Shanghai Macklin Biochemical Co., Ltd. (Shanghai, China). Absolute ethanol (C_2_H_5_OH) was supplied by Xilong Science Co., Ltd. (Shantou, China). Chitin (biopolymer grade), Fe_3_O_4_ nanoparticles (20–30 nm, 99.9%), ciprofloxacin (CIP, ≥98%) and nitroblue tetrazolium chloride (NBT, ≥98%) were obtained from Aladdin Chemistry Co., Ltd. (Shanghai, China). sodium hydroxide (NaOH, AR) was purchased from Tianjin Fortuna Chemical Co., Ltd. (Tianjin, China). All solutions were prepared using ultrapure water (18.2 MΩ·cm at 25 °C) purified via a two-stage ion-exchange and reverse osmosis system (Milli-Q Advantage, Millipore, Burlington, MA, USA).

### 3.2. Fabrication of Chitin/BiOCl Composites

A precursor solution was prepared by dissolving 8.0 mmol of Bi(NO_3_)_3_·5H_2_O and KCl in 60 mL of ethylene glycol (EG) under continuous stirring until complete dissolution. Subsequently, 20 mL of a chitin solution (chitin was dissolved in a mixed solution of sodium hydroxide and urea under low-temperature conditions; this method was reported in relevant literature [26]) with varying concentrations (0.5, 1.0, 1.5, 2.0, or 2.5 wt%) was added to the mixture. After stirring for 30 min, the homogeneous solution was transferred into a 100 mL polytetrafluoroethylene (PTFE)-lined autoclave and heated at 120 °C for 12 h in a drying oven, followed by natural cooling to room temperature. The resulting precipitates were thoroughly washed with deionized water and ethanol through centrifugation and dried at 60 °C for 12 h. The obtained samples were labeled as y-chitin/BiOCl, where y denotes the weight percentage of chitin.

### 3.3. Fabrication of Fe_3_O_4_/Chitin/BiOCl Composites

To optimize Fe_3_O_4_ loading, the chitin content was fixed at 2.0 wt% (determined as optimal in prior tests). Various amounts of Fe_3_O_4_ nanoparticles (5, 10, 15, 20, or 25 wt%) were ultrasonically dispersed into the precursor solution for 30 min. The subsequent steps—hydrothermal treatment, washing, and drying—were identical to those described in Section 2.1. The final products were designated as x-Fe_3_O_4_/2%chitin-BiOCl, where x represents the weight percentage of Fe_3_O_4_.

### 3.4. Characterizations

X-ray diffraction (XRD) patterns were recorded using a Bede D1 System diffractometer (Bede Scientific Instruments Ltd., Durham, UK) with Cu-Kα radiation (λ = 1.5406 Å). Morphological and elemental analyses were conducted with a field-emission scanning electron microscope (FE-SEM, JSM-6701F, JEOL Ltd., Tokyo, Japan) equipped with energy-dispersive X-ray spectroscopy (EDS). Transmission electron microscope (TEM) images were obtained via a FEI TF20 transmission electron microscope. Surface area and porosity measurements were carried out via nitrogen adsorption–desorption isotherms at 77 K using a JW-BK132F analyzer (JWGB Sci. & Tech. Co., Ltd., Beijing, China). The UV-Vis diffuse reflectance spectra were investigated on a TU-1900 spectrophotometer using BaSO_4_ as a reference and were converted from reflection to absorbance by the Kubelka–Munk method. Fourier-transform infrared (FT-IR) spectra were recorded at room temperature with a KBr pellet on a Nicolet 5700 spectrometer. UV-vis absorption spectra were measured using a UV-3600 Plus spectrophotometer (Beijing Purkinje General Instrument Co., Ltd., Beijing, China). The magnetic property was examined by a vibrating sample magnetometer (VSM) using Lake Shore 7404 apparatus at 25 °C with a magnetic field of ±30,000 Oe.

### 3.5. Photocatalytic Experiments

The photocatalytic activity of the obtained products was investigated by degrading the pale yellow ciprofloxacin (CIP) solution using a 300-W Xe-lamp with a 420 nm cut-off filter. In a typical experiment, 0.10 g of the photocatalyst was dispersed into 250 mL of CIP aqueous solution (10 mg/L) within a quartz reactor. Prior to irradiation, the suspension was magnetically stirred in the dark for 40 min to establish adsorption-desorption equilibrium. Subsequently, the mixture was exposed to visible light (λ ≥ 420 nm) with continuous stirring for 120 min. Aliquots (5 mL) were collected at 30-min intervals and immediately filtered through a 0.22 μm aqueous membrane filter to remove residual catalyst particles. The CIP concentration in the filtrate was quantified via UV-vis spectrophotometry by measuring the absorbance at λ = 276 nm.

The photocatalytic degradation efficiency was calculated as follows:

Remove = (1 − C/C_0_) × 100 (5)

where C_0_ and C are the initial and retained concentrations of CIP after adsorption equilibrium.

The degradation kinetics of CIP followed a pseudo-first-order reaction model [27], expressed as:

ln (C_0_/C) = kt (6)

where k (min^−1^) is the apparent rate constant, and t (min) is the irradiation time.

### 3.6. Determination of Active Species

To identify the reactive species involved in the catalytic mechanism, radical scavenging experiments were conducted. Specifically, 1.0 mmol/L isopropyl alcohol (IPA, AR) and 1.0 mmol/L triethanolamine (TEOA, AR) were introduced into the reaction system to quench hydroxyl radicals (·OH) and holes (h^+^), respectively. Additionally, nitroblue tetrazolium (NBT, 2.5 × 10^−5^ mol/L) was employed to quantify superoxide radicals (·O_2_^−^) by monitoring its characteristic absorption at λ = 259 nm. In these experiments, NBT replaced ciprofloxacin (CIP) as the probe molecule, while all other procedures—including dark adsorption, irradiation, and sampling remained consistent with the photocatalytic degradation protocol described in Section 2.

## 4. Conclusions

In this study, a magnetically recoverable visible-light-responsive photocatalyst, Fe_3_O_4_/chitin/BiOCl, was successfully synthesized and optimized. Comprehensive characterizations via TEM, X-ray diffraction (XRD), and X-ray photoelectron spectroscopy (XPS) confirmed that chitin integration extends the visible-light absorption range of BiOCl. The composite exhibited enhanced specific surface area and porosity, which improved pollutant adsorption capacity, while suppressed electron–hole recombination synergistically boosted photocatalytic activity.

The incorporation of Fe_3_O_4_ enabled efficient magnetic recovery under an external magnetic field. Remarkably, the catalyst retained ~ 85% degradation efficiency after five consecutive cycles, with no structural alterations observed in post-reaction XRD analysis, demonstrating exceptional stability. Furthermore, the composite displayed superior photocatalytic performance toward both single and mixed pollutants under visible light. Systematic optimization of reaction parameters identified ideal degradation conditions. Radical trapping experiments verified that h^+^, ·OH, and ·O_2_^−^ serve as the dominant active species.

This work provides a practical framework for designing magnetically separable visible-light photocatalysts, offering significant potential for sustainable organic pollutant remediation.

## Data Availability

The original contributions presented in this study are included in the article. Further inquiries can be directed to the corresponding authors.
